# Is episodic future thinking effective in mitigating the influence of time preference in time trade-off?

**DOI:** 10.1007/s10198-025-01812-4

**Published:** 2025-09-12

**Authors:** Zhongyu Lang, Liying Zhang, Stefan A. Lipman, Bradley Sugden, Kim Rand, Arthur E. Attema

**Affiliations:** 1 Erasmus School of Health Policy & Management, Erasmus University, P.O. Box 1738, 3000 DR Rotterdam, the Netherlands; 2 Erasmus Centre for Health Economics Rotterdam (EsCHER), Rotterdam, the Netherlands; 3 Health Services Research Centre, Akershus University Hospital, Akershus, Norway; 4 Math in Health B.V, Klimmen, the Netherlands; 5 Department of Clinical Epidemiology and Medical Technology Assessment, Maastricht University Medical Centre, Maastricht, the Netherlands

**Keywords:** EQ-5D-5L, Time preference, Time trade-off, Discounting, QALYs, Episodic future thinking

## Abstract

**Objectives:**

The composite time trade-off (cTTO) method has been found to be influenced by time preferences for future life years, which typically results in a downward bias on cTTO utilities without adjustment. Contrary to prior research that adjusted for this distortion ex-post, this study takes an ex-ante approach, using Episodic Future Thinking (EFT), to potentially prevent time preference distortion. We aim to investigate the effect of EFT on time preference and cTTO utilities compared to using alternative methods.

**Methods:**

A total of 150 participants from the UK general public were recruited for interviewer-led online interviews and randomly assigned to either the control or treatment group. In the control group, they were asked to recall recent memories using the Episodic Recent Thinking (ERT) protocol, serving as filler tasks; in the treatment group, they were asked to imagine life in the next 10 to 20 years, i.e. using an EFT protocol. Afterwards, respondents were asked to value seven EQ-5D-5L health states with cTTO tasks, followed by a nonparametric method to measure time preference.

**Results:**

We observed a similar pattern of time preference across the two groups, with the majority discounting positively. EFT did not significantly affect time preference. In addition, the difference between cTTO utilities mitigated by EFT and those adjusted using the ex-post approach for time preference is minimal.

**Conclusions:**

In conclusion, EFT does not seem to mitigate time preference for life years and has negligible effect on cTTO utilities, necessitating alternative strategies for reducing bias in health utilities.

## Introduction

The EuroQol-5D (EQ-5D) is a measure of health-related quality-of-life [21, 30], which describes health states in terms of five dimensions: mobility, self-care, usual activities, pain/discomfort, and anxiety/depression [13]. Each dimension consists of three (EQ-5D-3L) or five (EQ-5D-5L) levels of severity [19]. Composite time trade-off (cTTO) and the Discrete Choice Experiment (DCE) represent the two most prominent valuation techniques for deriving health state utilities for EQ-5D [47, 57].

For cTTO, respondents are asked to imagine living 10 more years in an EQ-5D state and compare this to some years in full health (< 10). This process involves an iterative adjustment of duration of life in full health until respondents are indifferent between the two scenarios. If responses indicate the given health state is worse than dead (WTD) (i.e., respondent prefers immediate death to 10 years in the given health state), the lead-time TTO protocol is initiated. In that case, 10 Years of lead time in full health is added to the 10 years in the impaired health state. DCE methods can also involve such trade-offs on health states over different durations [9, 33].

Importantly, respondents’ choices in these tasks involving durations could be influenced by time preferences since individuals often tend to assign lower weights to future years, relative to earlier years [7, 11]. Consequently, the stronger this tendency is (i.e., the more they discount the future), the more years will be given up. The possibility of such non-linear time preferences implies that the cTTO may not accurately elicit preferences for health states with the conventional QALY model [28], which assumes a linear utility of life duration. Thus, it is recommendable to adjust for the distortion caused by time preferences to obtain less biased estimates of health state utilities [3, 29].

Several approaches have been proposed to correct utilities for time preferences, especially when the TTO technique is used. Unfortunately, most are accompanied by substantial disadvantages. One approach applies a uniform discount rate to all utilities [39], but it neglects considerable heterogeneity in individual time preferences [14]. A second approach is to measure individual time preferences and use such estimates to adjust cTTO utilities. For example, Martin et al. [40] adjusted time trade-off utilities based on the utility of life duration function from each patient and Van der Pol and Roux [60] suggested a metric for the utility of lifespan by gauging how individuals assigned weights to different years for different health states. A nonparametric correction was adopted to adjust utility weights by eliciting prospect theory parameters for various health states [36]. Other studies used the direct method to adjust for time preference [7, 35], which has the advantages of not relying on parametric assumptions. Thirdly, it is possible to combine a lead-time with a lag-time protocol [7] or to use a standard (c)TTO with two different time horizons [23, 45], and to subsequently solve for the discount rate and utility that fit the answers to these two questions. Fourth, the DCE method can account for nonlinear time preferences by including a DCE design including duration that is specifically optimized to measure deviations from linear time preferences [29, 63]. However, all the aforementioned procedures require ex-post correction of the utilities, where the distorting influence of time preference is only addressed after it has affected cTTO/DCE responses. Additionally, as discussed, the distorting influence of time preferences (and as such, the effect of adjusting for them) may also impact health states considered better than dead (BTD) or WTD in opposite directions [35].

A procedure that successfully prevents or ameliorates time preference distortion ex-ante might therefore be preferable, as it does not rely on potentially error-prone measurement or estimation of time preferences. Episodic Future Thinking (EFT) may provide a feasible approach by which time preference distortion is reduced ex ante. EFT refers to a protocol, extensively validated in psychological and behavioral science research [2, 52, 53], that involves mentally simulating possible future events in detail. This simulation helps individuals pre-experience events and imagine themselves in a future scenario, considering various situational and emotional factors, in order to anticipate and make decisions about future outcomes [2]. In this context, EFT aims to improve the future-orientation of individuals and reframes the temporality of decision-making in the moment [46].

Empirical research suggests that EFT can effectively reduce time preference across various domains, including health-related behaviors, consumption choices, and financial decision-making [10, 44, 49]. Studies demonstrated that individuals who engage in EFT exhibit decreased delay discounting of future rewards [17, 49, 56, 59]. Furthermore, EFT has proven effective in improving health-related behaviors, such as reducing energy intake, promoting healthier snack choices, decreasing impulsive eating behaviors, reducing cigarette consumption and increasing motivation to quit smoking [12, 17, 18, 41, 46, 55, 56, 59]. These findings highlight the potential of EFT as a viable strategy for reducing time preference distortion ex-ante.

In light of this background, this study aims to explore the potential of EFT in mitigating the distortion associated with time preference in cTTO tasks. We conducted an experiment employing a newly designed EFT manipulation in health state valuation. Subsequently, respondents were required to complete cTTO tasks including tasks used for an ex-post approach for adjusting the time preference. We investigate the extent to which promoting EFT can reduce the distortion related to time preference in cTTO valuation, and whether EFT could serve as an alternative to adjusting for time preference ex-post by comparing the cTTO utilities between EFT and the ex-post method.

## Methods

### Sampling and data collection

This study received approval from the research Ethics Review Committee of Erasmus School of Health Policy and Management (ETH2122-0403). A total of 150 participants were recruited from the UK population for the experiment through an online platform, Prolific. Respondents were asked to participate in personal online video interviews, which lasted for a maximum of 1 h. After completion, they were rewarded 10 GBP. The data collection was performed between June and August 2022. All interviews were conducted digitally by a team of two trained interviewers, using interviewing software programmed in R Shiny. The software was run locally on interviewers’ PC who shared their screens with the video conferencing application Zoom. Lipman 34 suggested that, compared to in-person interviews, technology-assisted interviews provide increased flexibility and cost efficiency, benefiting both participants and interviewers. Note that before the main study, we conducted three pilot tests with colleagues to ascertain the robustness of the Shiny software used in the main experiment.

The chosen sample size was based on the budget constraints and prior studies on the similar topic. EQ-5D value sets are typically based on approximately 100 observations per health state [65]. Furthermore, our sample size is similar to an earlier work [35] on adjusting for time preference in cTTO. In addition, for previous EFT studies, most of the research, with sample sizes typically not exceeding 50 for each treatment or control group, found significant effects of EFT in various domains, such as reducing time preference or energy intake [17, 18, 41, 46]. Moreover, we conducted a power analysis to ensure the adequacy of our sample size. As there is no prior research specifically examining the effect of EFT on cTTO utilities to reference, we used the average effect size (Hedges’ *g* = 0.52) from a meta-analysis on the effect of EFT on time preference [67]. Based on this desired estimate, we calculated that a minimum of 59 participants per group would be required to detect a similar effect of EFT on time preference adjustment, with 80% power at a 5% significance level (see Appendix 9).

### Health states

Respondents in this study were asked to value health states selected from the EQ-5D-5L system [25]. EQ-5D-5L includes five dimensions—mobility, self-care, usual activities, pain/discomfort, and anxiety/depression—with five levels of problems: no problems, slight problems, moderate problems, severe problems, and extreme problems. In general, a combination of five numbers represents a specific health state, where each number indicates the level of problems experienced in a dimension. For example, 11342 refers to no problems with mobility, no problems with self-care, moderate problems with doing usual activities, severe problems with pain or discomfort, and slight problems of being anxious or depressed.

In this study, we selected seven EQ-5D-5L health states (11221, 54231, 34515, 35245, 33333, 45144, and 55555) to encompass a broad spectrum of health conditions [68] within our practical constraints of time and budget^1^ Our selected profiles range from relatively good health (11221), through moderately impaired health (33333), to extremely severe impairment (55555). We also referred to prior research on the EQ-5D-5L value set [20] to ensure our selection included both BTD and WTD states and cover a wide range of utility values. In addition, we ensured that each of the five EQ-5D-5L dimensions is represented at level 5 in at least one selected profile. This heterogeneity across dimensions enables us to examine whether EFT effects vary by the dimension-specific severity of the health state.

### Design

**Fig. 1:**
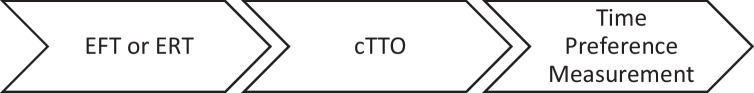
Timeline of the experiment (three blocks in total)

Our study consists of three blocks, as illustrated in the Fig. 1. In the first block, to investigate the effect of EFT on cTTO utilities, we conducted a between-subject experiment in which participants were randomly assigned to either the treatment (EFT) or control (ERT) group. In the treatment group, interviewers instructed participants to engage in an EFT task, imagining their future life in the next 10 and 20 years with a specific health state 33333. Participants described the imagined future life including how it would affect their mobility, self-care, usual activities, and physical and mental well-being, as vividly as possible, such as describing their work status, family, feeling of experiencing health problems.

The selection of health state 33333—moderate problems in mobility, self-care, usual activities, pain/discomfort, and anxiety/depression was to trigger detailed imagination about future constraints yet was generic enough to allow participants to personalize their envisioned scenarios according to their own life contexts, such as work, home activities, and emotional experiences. We aimed to balance the need for a standardized experimental anchor with sufficient flexibility for participants to create personally meaningful and specific future scenarios.

In the control group, participants were instructed to perform an Episodic Recent Thinking (ERT) task, recalling details of recent events, such as meals eaten, recent activities, and experiences during the last hour. In both groups, the interviewers were instructed to ask the respondents to describe more details if they evaluate their descriptions are not vivid enough. The ERT task was designed to be unrelated to the health valuation tasks but served as a filler task to ensure both the treatment and control group spend a similar amount of time and effort in block 1. Moreover, previous research suggests that the effect of EFT is more pronounced when using ERT as the control task compared to having no task [67]. After the EFT or ERT tasks, participants in both groups rated the vividness of the events they described.

In the second block, respondents from both groups were asked to complete cTTO tasks for seven health states. In the final block, all the respondents were asked to complete time preference measurement task with the health state 33333. Further details on the cTTO tasks and the method used for time preference measurement can be found in sections cTTO operationalization and Time preference.

### Procedure

At the start of the survey, each participant was asked to provide basic demographic information, including their education level, age, gender, family size, marital status, whether they were religious, and subjective life expectancy (SLE). Next, they were asked to self-report their health using EQ-5D-5L and on a Visual Analogue Scale (EQ-VAS), from 0 (worst imaginable health state) to 100 (best imaginable health state). All the specific questions are included in Appendix 1, as well as a link to the whole design.

In the next stage, after being assigned to either of the EFT or ERT groups, participants followed the instruction and described future (EFT) or recent (ERT) life events accordingly. The EFT group created personalized episodic future simulation cues in two steps. They were asked to imagine their life 10 and 20 years into the future in a specific health state (33333), constructing a vivid future scene through memory and imagination. First, participants answered a series of "What–With Whom–Where"questions to elaborate on the details of a future event, along with a "How are you feeling?" question to capture their emotions and feelings. Next, participants were asked to rate their description of the event on a 5-point scale Likert scale for vividness. The ERT group generated episodic recent thinking cues through a procedure similar to the EFT group, first describing an event that occurred on the morning of the experimental day and then rating its vividness. Moreover, for both groups, if participants’ descriptions were not sufficiently detailed or specific, the experimenter could ask additional questions to guide the process and enhance the vividness. More detailed information regarding the questions asked in the EFT and ERT groups is given in Appendix 2.

Then, each respondent was directed to the interface of the Shiny software to complete the cTTO tasks for seven health states, which were presented in random order. Afterward, participants completed the time preference task.

### cTTO operationalization

Following the EQ-VT protocol [47, 48] the cTTO method was implemented with a conventional 10-year duration. Respondents are asked to consider living in a described health state for 10 years, followed by immediate death, and to compare this with living a certain number of years in full health. cTTO combines the conventional TTO and lead-time TTO [27]. Conventional TTO questions are used to value health states better or equal to being dead. If respondents prefer immediate death to living for 10 years in the described health state, it suggests they consider the health state WTD. In such cases, respondents are directed to answer the lead-time TTO questions, which involves adding 10 years in full health before the 10 years in the WTD health state, resulting in a total of 20 years.

In the conventional TTO, the utility of a health state $Q$, denoted as $H(Q)$, is elicited by asking interviewees to make a series of choices comparing 10 years in $Q$, with $Y$ years in full health ($FH$), until an indifference$, (Q,10)\sim (FH,Y)$, is found. According to the general QALY model [42] this indifference is evaluated as:

1 $$H(Q)*L(10)=H(FH)*L(Y)$$

In this equation, the utility function $L(T)$ represents the utility of a life duration of $T$ years. $H(Q)$ ranges between 0 (death) and 1 (full health). As usual, $H(FH)$ is anchored at 1, such that Eq. (1) can be solved for $H(Q)$ as follows:

2 $$H(Q)=\frac{L(Y)}{L(10)}$$

The conventional QALY model assumes a linear model for the utility of life duration, which implies $L(Y)=Y$. Then the Eq. (2) simplifies to $H(Q)=\frac{Y}{10}$.

As mentioned above, the lead-time TTO method is used to measure WTD health states, where 10 years in full health is followed by 10 years in $Q$. The indifference searched for is: $(FH,10;Q,10)\sim (FH,Y)$, i.e. one needs to compare a period living for $X$ Years in full health to a period starting with 10 Years in full health, followed by 10 years in $Q$, until reaching an indifference between these two periods. According to the general QALY model, this indifference is evaluated by:

3 $$H(FH)*L(10)+ H(Q)*[L(20)-L(10)]=H(FH)*L(Y)$$

where $H(FH)$ is again equal to 1. Solving for $H(Q)$ yields:

4 $$H(Q)=\frac{[L(Y)-L(10)]}{[L(20)-L(10)]}$$

In the linear model, this becomes $H(Q)=\frac{(Y-10)}{10}$.

As described by [32], time preferences can be captured through the utility function $L(T)$ and evaluating cTTO utilities with Eq. (2) and (4) allows for correction for time preference, as opposed to the linear approach.

### Time preference

There are two categories of discounting in our study. Positive discounting means that individuals tend to value future life years less than present, meaning they prefer to receive health benefits sooner than later. Conversely, negative discounting refers to future life years being valued more than present ones. No discounting implies no preference between present and future life years. We used the direct method to measure time preference, which has been successfully implemented to adjust TTO utilities in previous studies [4–6, 35]. This method requires respondents to choose between two scenarios, each involving a transition between two health states: one better and one worse. The key difference between the scenarios is the sequence of health states, while the total time spent in each remains constant. The goal is to identify whether respondents prefer to experience better health earlier or later, as well as how many years in good health they are willing to sacrifice to achieve that preference.

**Fig. 2:**
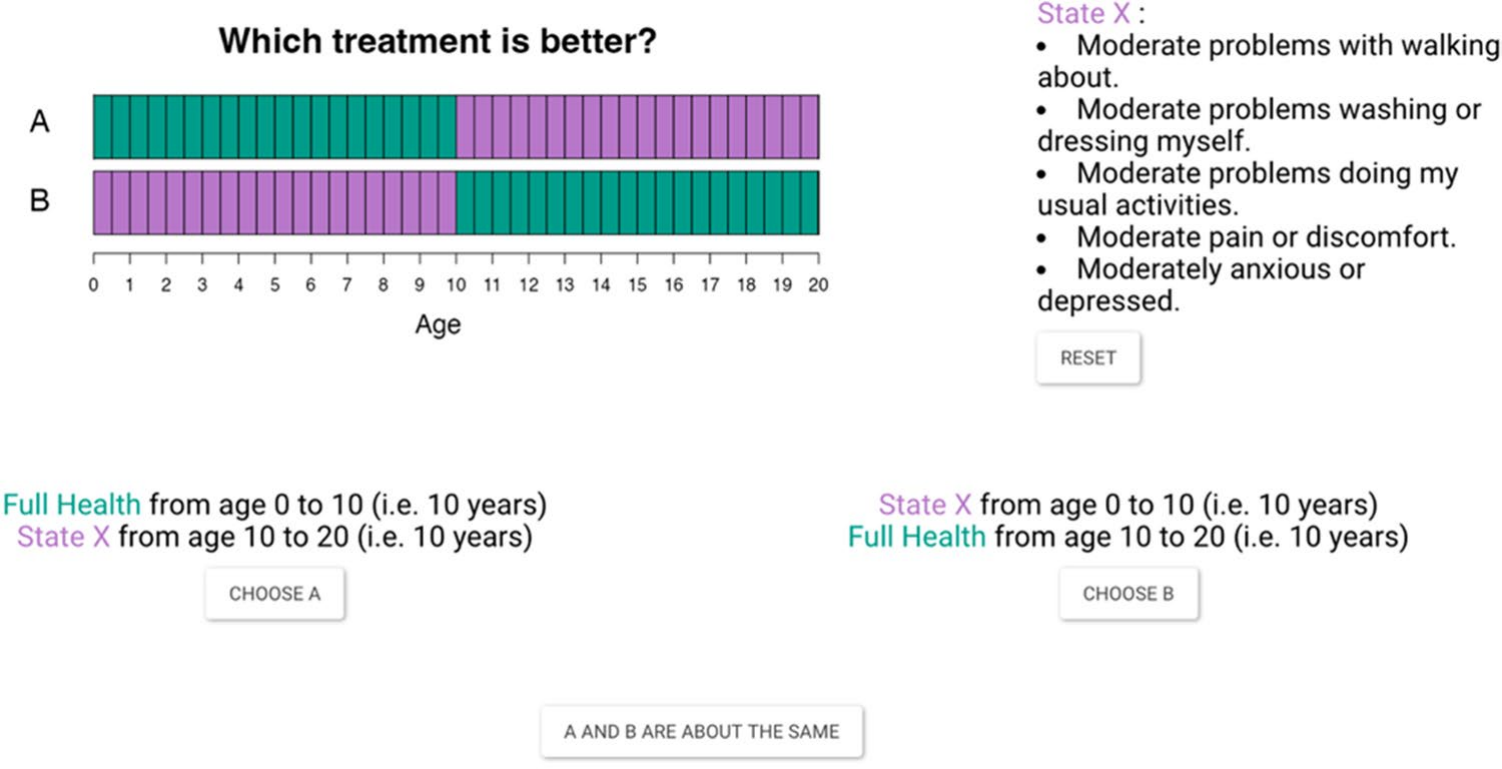
Screenshot of the task of time preference measurement

We set the total time frame to 20 years in both scenarios, matching the maximum duration in the cTTO task when the lead-time component was introduced. Time preference was measured between $L(0) = 0$ and $L(20) = 1$. Figure 2 presents the starting point of this task, with two health states involved: 33333 (State $X$) and 11111 (Full Health, $FH$).

1. Option A: 10 years in full health, followed by 10 years in State $X$.
2. Option B: 10 years in State $X$, followed by 10 years in full health.

Participants were informed that their health status beyond the 20-year period was unknown in both options. The indifference point (${T}_{1/2}$)—where respondents perceived the two sequences as equal—was elicited using the following equation:

$$[(FH, 0-{T}_{1/2}; X, ({T}_{1/2}-20)] \sim [X, 0-{T}_{1/2}; FH, ({T}_{1/2}-20)]$$

Following the general QALY model, we then obtain:

5 $$L\left({T}_{1/2}\right)*H\left(FH\right)+\left[L\left(20\right)-L\left({T}_{1/2}\right)\right]*H\left(X\right)=L({T}_{1/2})*H(X)+[L(20)-L({T}_{1/2})]*H(FH)$$

This can be rearranged based on $H(FH)=1$, as follows:

6 $$L({T}_{1/2})=L(20)-L({T}_{1/2})$$

Given that $L(20) = 1$, it follows that $L({T}_{1/2}) = 0.5$.

Using this approach [8], we can extend the measurement to other time points on the discount function, allowing us to derive:

$$L\left({T}_\frac{1}{8}\right)= 0.125; L\left({T}_\frac{1}{4}\right)= 0.25; L\left({T}_\frac{1}{2}\right)= 0.5; L\left({T}_\frac{3}{4}\right)= 0.75; L\left({T}_\frac{7}{8}\right)= 0.875.$$

These points enabled interpolation to approximate the utility of life duration at other time intervals if needed. We then used a non-parametric approach, area under the curve (AUC), to describe time preference: AUC > 0.5 (Positive discounting); AUC < 0.5 (Negative discounting); AUC = 0.5 (No discounting). An example of how the direct method was used to adjust cTTO utilities for time preference is provided in Appendix 3.

### Analysis strategy

Prior to conducting the main analyses, we explored if randomization was successful by comparing demographics between the treatment and control group. We examined the data quality and compared the responses between the treatment and control groups to identify any potentially problematic responses. The data quality was assessed by comparing several indicators provided by previous studies [26, 37].

For the primary analyses, we investigated the influence of EFT on mitigating time preference in cTTO by comparing individuals’ time preference between the treatment and control groups elicited through the tasks in the final block. Mixed-effect regressions of cTTO utilities on the EFT treatment and covariates were utilized to explore potential differences in cTTO utilities between the ERT and EFT groups. Furthermore, we compared the cTTO utilities adjusted for time preferences using two different approaches, i.e., ex-ante adjustment with the EFT and ex-post adjustment with the direct method, to evaluate whether EFT yields similar cTTO utilities to the ex-post time preference adjustment. This comparison enables us to assess whether adjusting for time preferences before cTTO tasks has similar effectiveness as adjusting for them after cTTO data collection in influencing cTTO utilities.

## Results

### Sample description

Table 1 summarizes sample characteristics for the ERT and EFT groups. The randomized assignment resulted in balanced samples with no significant demographic differences between the ERT and EFT groups (Chi squared tests, all p’s > 0.14). In both groups, the majority of participants, over 64%, were aged between 18 and 44 years, with a higher proportion of individuals over 65 years old in the ERT group. The sample was highly educated: more than 65% of participants in both groups received higher education, the criteria for this classification can be found in Appendix 1. Approximately 54% of the overall sample was female, and about 30% identified as religious. Subjective life expectancies were similar across both groups, averaging around 82 years. The mean vividness scores in both tasks were significantly different between two groups at 1% level (T-test, *p* = 0.008 and *p* < 0.001).

**Table 1:** Summary statistics of the sample by groups

Characteristics	All participants (n = 150)	ERT group (n = 78)	EFT group (n = 72)
Age, n (%) 18–44 45–64 65 +	102 (68%) 39 (26%) 9 (6%)	50 (64.1%) 22 (28.2%) 6 (7.7%)	52 (72.2%) 17 (23.6%) 3 (4.2%)
Education, n (%) Higher education Middle-level education Lower education	104 (69.3%) 28 (18.7%) 18 (12%)	52 (66.7%) 15 (19.2%) 11 (14.1%)	52 (72.2%) 13 (18.1%) 7 (9.7%)
Gender, n (%) Male Female	68 (45.3%) 82 (54.7%)	36 (46.2%) 42 (53.8%)	32 (44.4%) 40 (55.6%)
VAS: Health status, mean (SD)	76.81 (17.67)	76.4 (18.99)	77.25 (16.25)
Subjective life expectancy, mean (SD)	81.99 (10.42)	81.87 (11.64)	82.11 (8.98)
Having children, n (%)	47 (31.5%)	22 (28.2%)	25 (34.7%)
Being religious, n (%)	46 (30.7%)	25 (32.1%)	21 (29.2%)
Married, n (%)	55 (36.7%)	30 (38.5%)	25 (34.7%)
Vividness-1	3.58	3.81	3.33
Vividness-2	3.79	4.17	3.38

Lower education: elementary school or pre-vocational secondary education; middle-level education: secondary vocational education or upper-level secondary school); higher education: higher professional education or university; vividness-1: the vividness score for the first task; vividness-2: the vividness score for the second task. Both vividness ranges from 1 (not vivid) to 5 (very vivid)

### Data quality

As summarized in Table 2, the data quality in the control group (n = 78) and the treatment group (n = 72) are listed separately. Non-trading responses ($H(Q)=1$), mean that respondents do not trade-off life years and they do not sacrifice longevity for quality of life; all-in-trading responses ($H(Q)=-1$), represent participants trade-off all life years, meaning they are willing to give up all life years to avoid living in the impaired health state; and zero responses ($H(Q)=0$), mean that people are indifferent between living in the impaired health and immediate death, resulting in a utility of zero. All three showed a similar pattern across both groups. No significant differences were observed between the EFT and ERT groups (Chi-square test, all p’s > 0.15). Overall, the data quality is comparable to that of a previous study [37], with few strong dominance violations (utilities of strictly worse health states are higher than utilities of strictly better health states), ranging between 3.2% and 4.9%.

**Table 2:** Data quality for both control and treatment groups

Categories	Control group (ERT) n = 78 (Out of 150)	Treatment group (EFT) n = 72 (Out of 150)	Total n = 150
Non-trading responses ($H(Q)= 1$)	40 (7.3%)	44 (8.7%)	84 (8%)
All-in-trading responses ($H(Q)= -1$)	72 (13.1%)	60 (11.9%)	132 (12.6%)
Zero responses ($H(Q)=0$)	36 (6.6%)	34 (6.7%)	70 (6.7%)
Respondents without negative utilities	34 (43.6%)	35 (48.6%)	69 (6.6%)
Respondents without 0.5-year increments	8 (10.3%)	10 (13.9%)	18 (1.7%)
Weak dominance violation (e.g., u(hs35245) < = u(hs55555))	119 (15.3%) (Out of 780)	99 (13.8%) (Out of 720)	218 (14.5%) (Out of 1500)
Strong dominance violation (e.g., u(hs35245) < u(hs55555))	38 (4.9%) (Out of 780)	23 (3.2%) (Out of 720)	61 (4.1%) (Out of 1500)

150 respondents in total, × 7 health states in total; 78 respondents in the control group, 546 observations; 72 respondents in the treatment group, 504 observations; 78/72 respondents × 10 health state pairs for weak/strong dominance

### Time preference

Table 3 below shows the mean estimate of the area under the curve (AUC) and classification of respondents in both groups based on their time preference, as measured by the direct method. Additional average AUC values of two time preferences in two groups are provided in Appendix 8. The majority in both groups discounted positively, only a few exhibited neutral time preference (i.e., no discounting), and slightly fewer participants discounted negatively in the EFT group than in the ERT group. However, the Binomial proportion test showed no difference in all three classifications of discounting between the two groups, with p values of 0.83, 0.39 and 0.42, respectively. No statistically significant difference was observed in the mean AUC values (T-test, p = 0.68). These results indicate that EFT has no overall effect on individuals’ discounting of future life years.

**Table 3:** Classification of respondents in both groups according to their time preference

	Control group (ERT) n = 78	Treatment group (EFT) n = 72
Positive discounting (i.e., future life years decrease in value)	64 (82.1%)	60 (83.3%)
Negative discounting (i.e., future life years increase in value)	11 (14.1%)	6 (8.3%)
No discounting	3 (3.8%)	6 (8.3%)
Mean AUC	0.57	0.57

### cTTO utilities

The distribution and variance of cTTO utilities were similar in both ERT and EFT groups, shown in Appendix 4, indicating EFT did not influence cTTO utility elicitation. After adjusting for time preference, distributions remained similar, but the values were no longer censored at −1 due to the removal of the linearity assumption after applying corrections. Results presented in Table 4, from the independent sample t-test, confirmed the insignificant effect of EFT since no difference was found between the two groups regarding the unadjusted cTTO utilities (t-test all p’s > 0.16) and adjusted cTTO utilities (t-test all p’s > 0.06). By using paired t-tests to compare utilities before and after adjusting for time preference within each group, we found statistically significant difference in most health states, suggesting that post-hoc adjustment for time preference, using estimates derived through the effect of direct method, influences cTTO.

**Table 4:** Mean cTTO and adjusted cTTO utilities (standard deviations) for all states

State	Unadjusted cTTO (ERT)	Adjusted cTTO (ERT)	Unadjusted cTTO (EFT)	Adjusted cTTO (EFT)	BTD percentage
11221	0.84 (0.20)	0.85 (0.20) ***	0.88 (0.16)	0.89 (0.15) *	99.33%
54231	0.39 (0.49)	0.41 (0.57)	0.35 (0.49)	0.35 (0.59)	82.67%
34511	0.11 (0.59)	−0.29 (1.92) **	0.22 (0.58)	0.17 (0.77) *	71.33%
35245	−0.02 (0.64)	−0.47 (1.95) **	0.09 (0.61)	−0.07 (1.15) *	60.67%
33333	0.53 (0.45)	0.55 (0.50)	0.63 (0.39)	0.65 (0.45)	90.67%
45144	−0.01 (0.60)	−0.34 (1.59) **	0.01 (0.61)	−0.19 (1.17) **	60%
55555	−0.34 (0.58)	−0.92 (1.87) ***	−0.3 (0.58)	−0.59 (1.18) ***	32%

* indicates significant difference between unadjusted and adjusted cTTO utilities within ERT or EFT group at the 10% level; ** indicates significant difference at the 5% level; *** indicates significant difference at the 1% level

### Regression

Next, we conducted a mixed effects regression (MER) to explore whether EFT has significant influences on the cTTO utilities. The table below includes three models at an aggregated level. Model 1 included only the EFT treatment dummy and health states, and model 2 incorporated additional demographic variables. No significant treatment effect of EFT was found. Individuals with a higher education level demonstrated significantly higher cTTO utilities compared to those with lower education. However, no significant difference in cTTO utilities was observed between the lower and middle education groups. Males, married individuals, and non-religious individuals had significantly higher cTTO utilities compared to females, unmarried individuals, and religious individuals, respectively. Individuals with better self-reported health were willing to trade more years in the cTTO tasks, although the effect size was small.

We also investigated two variables describing the self-reported vividness with which individuals thought about the ERT or EFT events; both were found to be insignificant. Furthermore, we examined whether EFT yielded similar cTTO utilities to those obtained through adjusting for time preference by creating a new utility variable in Model 3. In this model, the outcome variable combined discounting-adjusted cTTO utilities for the ERT group with the unadjusted cTTO utilities for the EFT group. Again, no significant differences in cTTO utilities were found between the EFT group and the ERT group adjusted for time preference (Table 5).

**Table 5:** Mixed effects regression of utilities, control, or treatment group with other demographic variables

cTTO utilities	1 (Unadjusted cTTO)	2 (Unadjusted cTTO)	3 (Utilities)
Treatment (EFT) Health state 11221 Health state 54231 Health state 34515 Health state 35245 Health state 33333 Health state 45144 Higher education	0.054 (0.064) 1.179 (0.042) *** 0.698 (0.042) *** 0.485 (0.042) *** 0.359 (0.042) *** 0.902 (0.042) *** 0.323 (0.042) ***	−0.0145 (0.072) 1.173 (0.042) *** 0.696 (0.042) *** 0.488 (0.042) *** 0.361 (0.042) *** 0.898 (0.042) *** 0.325 (0.042) *** 0.163 (0.097)	0.136 (0.135) 1.433 (0.085) *** 0.958 (0.085) *** 0.580 (0.085) *** 0.424 (0.085) *** 1.156 (0.085) *** 0.453 (0.085) *** 0.242 (0.180)
Middle level education		−0.068 (0.114)	0.049 (0.211)
45–64 years 65 + years Gender Whether having children		0.020 (0.070) −0.032 (0.140) 0.155 (0.062) ** −0.006 (0.074)	0.083 (0.131) −0.305 (0.260) 0.234 (0.115) ** −0.006 (0.137)
Marital status Religion		0.145 (0.073) ** −0.126 (0.070) *	0.248 (0.135) * −0.137 (0.129)
EQVAS		−0.003 (0.002) *	0.000 (0.003)
Subjective life expectancy		0.003 (0.003)	0.007 (0.006)
Vividness-1 Vividness-2		−0.025 (0.037) −0.289 (0.301)	−0.084 (0.082) −1.237 (0.560)

* indicates significance level at 10%; **indicates significance level at 5%; *** indicates significance level at 1%

Model 3 is the mixed effect regression with adjusted cTTO in ERT group and cTTO in EFT group as a robustness check

The reference health state is 55555

Vivideness-1/−2: two questions to test vividness of EFT or ERT manipulation, details can be found in Appendix 2

### Equivalence analysis

Given that both the t-tests and MER showed no significant effect of EFT on cTTO utilities, we further conducted post-hoc equivalence tests using the Two One-Sided Tests (TOST) procedure [31] to assess whether the differences in cTTO utilities between EFT and ERT, as well as between EFT and the ex-post adjustment method, were sufficiently small to be considered practically equivalent.

The TOST procedure is first needed to specify upper and lower equivalence bounds ($\pm\Delta$) based on the smallest effect size of interest (SESOI). It tests two composite null hypotheses: ${H}_{01}:\theta \le -\Delta$ and ${H}_{02}:\theta \ge\Delta$, where $\theta$ represents the effect or mean difference between the two groups that is being tested for equivalence. If both null hypotheses are rejected, the observed effect is statistically confirmed to lie within the equivalence bounds ($-\Delta$< $\theta$< $\Delta$), indicating that it is sufficiently close to zero to be considered practically equivalent (see Appendix 10).

We conducted two separate equivalence tests for cTTO utilities. First, we compared the mean cTTO utilities between the EFT and ERT groups. Second, we compared the cTTO utilities in the EFT group with the time preference–adjusted cTTO utilities in the ERT group, to evaluate the relative effectiveness of EFT and direct method. We separately defined two equivalence margins based on different criteria: (i) Δ = 0.074, corresponding to the minimally important difference (MID) for EQ-5D utility scores, which indicates a practically meaningful change [66]; and (ii) Cohen’s d = 0.20, a general rule of thumb for a small effect size [16], representing the smallest effect size of interest (SESOI) for detecting a meaningful difference between EFT and ERT. This second margin was converted to the cTTO scale by multiplying the effect size by the pooled standard deviation, yielding an equivalence margin of $\Delta = 0.20\times {SD}_{pooled}$.

Panel A in Table 6 shows that the first equivalence on cTTO utilities between EFT and ERT groups cannot be established under the strict MID threshold (± 0.074). The difference falls out of the upper bound and suggested that cTTO utilities influenced by EFT are higher than those in ERT group. However, when using the more conservative “small-effect” threshold (± 0.127), it allows us to conclude statistical equivalence between EFT and ERT on cTTO utilities. This finding aligns with our main result of no significant effect of EFT.

**Table 6:** Equivalence tests of cTTO utilities: EFT vs. ERT and EFT vs. direct method

Outcome	Equivalence bound (Δ)	90% CI for ${{{\theta}}}_{{{obs}}}$	*p***-**value ( ${{{H}}}_{01}:\boldsymbol{ }{{\theta}}$≤ − Δ)	*p*-value ( ${{{H}}}_{02}:{{\theta}}$≥ + Δ)	*Equivalent?*
*Panel A:* cTTO utilities (EFT vs ERT)	0.074 (MID, Walters & Brazier (2005))	[–0.011, 0.117]	< 0.001	0.296	No
	0.127 (Cohen’s *d* = 0.20 (“small”))	[–0.011, 0.117]	< 0.001	0.030	Yes
*Panel B:* Utilities (EFT vs. direct method)	0.074 (MID, Walters & Brazier (2005))	[0.176, 0.417]	< 0.001	> 0.999	No
	0.127 (Cohen’s *d* = 0.20 (“small”))	[0.176, 0.417]	< 0.001	0.790	No

${\theta }_{obs}= {\mu }_{EFT}- {\mu }_{ERT}$, where $\mu$ is the mean cTTO utilities in the responding group. In Panel A, ${\theta }_{obs}=0.053$. In Panel B, ${\theta }_{obs}=0.297$. The 90% confidence interval (CI) corresponds to α = 0.05 for each one-sided TOST. Utilities are constructed with adjusted cTTO in ERT group and cTTO in EFT group

In the second test, equivalence was not demonstrated under both criteria as shown in Panel B in Table 6. The inconsistency between the main findings and the equivalence test comparing cTTO utilities adjusted by EFT and by the ex-post method is not unexpected. First, equivalence testing involves two one-sided tests and requires stronger evidence to reject inequivalence, making it more conservative than standard regression when using the same type I error rate ($\alpha$). More importantly, whether two groups are considered equivalent depends on the equivalence margin selected. Moreover, in our study, the observed inequivalence may also be attributed to the large standard deviation of the cTTO utilities adjusted ex post for time preference (see Appendix 10). Therefore, we conclude that the difference in the impact of EFT and the direct method on mitigating time preference in cTTO utilities is minimal.

### Heterogeneity and sensitivity analysis

We conducted several sensitivity analyses to assess the robustness of our findings. We first analyzed better than dead and worse than dead observations separately (since they are affected by time preference in opposite directions) with demographic variables but found no difference, reported in Appendix 5. To investigate heterogeneous effects of EFT we performed additional regression analyses across different subgroups (reported in Appendix 6 and 7). We tested interaction effects between the following demographics including age, self-reported health, and vividness, based on the findings that EFT efficacy is associated with these factors [43, 50, 54, 67]. However, no significant interaction effects were found. We also examined whether EFT had specific impact on health state 33333 (used in the EFT manipulation), on the first health state respondents experienced (where EFT effects might be the strongest), and on the most severe health state 55555. Again, no significant effects of EFT were found in these subgroups (see details in Appendix 7).

## Discussion

This paper explored the potential of EFT in mitigating the distortion associated with time preference in cTTO tasks. As such, we compared cTTO utilities between an EFT group, which engaged in a task in which they vividly imagined their lives in the next 10–20 years before completing cTTO tasks for different health states, and an ERT group, which completed a filler task before the cTTO tasks commenced. Our findings revealed an insignificant influence of EFT on cTTO utilities, indicating that the process of eliciting respondents’ health utilities using the cTTO method may not be affected by EFT.

We found no evidence of a statistically significant effect of EFT on time preference, which conflicts with previously reported findings on the efficacy of EFT in reducing time preference [17, 49, 56, 59]. Recent meta-analyses have synthesized findings to report a moderate average effect in reducing discounting, primarily on monetary rewards, and to a lesser degree on health-relevant domains, such as food, smoking and alcohol [50, 67].^2^ Our research extends these studies to life years but observed no effect of EFT on the amount of discounting. The emotional valence, which is considered to contribute to the efficacy of EFT, may explain this outcome. Positive emotional experience elicited in EFT can effectively reduce discounting, while neutral and negative emotions do not [38]. In our study, subjects who are asked to imagine an impaired health state 33333 in the future might not evoke positive emotions to prompt themselves to weigh future life years more heavily. Moreover, previous research indicates that it can be difficult to report or predict health states over a period due to adaptation. For instance, participants might take declined mobility or the deterioration of usual activities as normal for the age in EFT, making it unclear how EFT influences time preferences for life years and predictions of chronic health states [64].

We also did not observe a significant effect of EFT on cTTO utilities. Besides the aforementioned possibilities leading to the inefficacy of adjusting time preference, the failure of EFT can also be due to the specific design of the EFT intervention. In our study, the episodic content might not have been as closely related to subsequent decisions as in previous research [44]. For example, earlier studies show that EFT reduces unhealthy eating only when the imagined future vividly involves eating‑ or weight‑related scenes; unrelated episodes fail to effectively reduce calorie intake [18, 55]. In our study, although participants were prompted to imagine life with limitations in all five EQ‑5D dimensions, constructing vivid, health‑specific episodes around an abstract profile such as 33333 may still have been difficult. Despite participants reporting relatively high vividness, we found no significant association between vividness and cTTO utilities. Moreover, we had no evidence suggesting whether the self-projection mechanism underlying EFT was effectively operated. Additionally, the effect of EFT on cTTO utilities for health state 33333 did not significantly differ from its effects on the other six health states, indicating that even closely matched episodic content does not necessarily ensure EFT effectiveness.

The effectiveness of EFT can also strongly depend on whether respondents had similar experiences before. For instance, Bromberg et al. [12] found no effect of EFT on alcohol consumption among healthy adolescents, contrasting with Mellis et al. [41] who reported a significant effect among current drinkers. Our largely healthy sample was unlikely to have lived through the impairments depicted, which may have weakened their emotional engagement and, in turn, the EFT’s effectiveness.

Apart from the design of EFT, the content of ERT may also affect the effectiveness of EFT. ERT, as the control condition, still asked respondents to engage in episodic thinking, albeit focused on the past rather than the future. Prior research suggests that engaging in episodic thought—whether about the past or future—can influence decision-making [53]. Thus, it is possible that the expected contrast between EFT and ERT was insufficient, as both tasks may have engaged similar cognitive processes relevant to time preference. These findings indicate that EFT may not be suitable for reducing discounting in cTTO, or that the intervention itself needs refinement. Future studies should consider asking participants to imagine the positive consequences of improved health and include a control condition that avoids episodic thinking, such as an attention matching task. In all, future experimental designs can be optimized by offering more vivid and health specific scenarios, adding manipulation check for self-projection and EFT effectiveness, using positive framing, and embedding content directly relevant to TTO decisions to create a clearer distinction between EFT and ERT. For example, integrating EFT cues into a coherent personal narrative, encouraging participants to envision how they wish to live their desired life instead of picturing an unfocused future, has been shown to strengthen the impact of EFT [22].

We also compared the effectiveness of adjusting for time preference in cTTO utilities using the ex-ante EFT approach and the ex-post direct method. The mixed-effect regression showed that cTTO utilities after EFT did not differ significantly from utilities adjusted with the direct time preference method. The direct method significantly influenced cTTO utilities by accounting for time preference, which aligns with most previous studies [4, 35, 37]. By contrast, there is no evidence that EFT influenced time preference and cTTO utilities. One explanation is EFT also influences cTTO utilities through other pathways in addition to time preference. Another might be attributed to a lower level of time preference and a higher proportion of negative time preference compared to previous studies [4, 24, 37]. The direct method, which measures time preference with sequences, may lead to low or even negative discounting when some individuals prefer improving sequences over deteriorating sequences [15]. Although a low level of discounting is not necessarily problematic and has also been observed with other elicitation methods [1, 14, 58], it indicates a systematically limited potential to reduce time preference. There is little to no evidence comparing discount rates systematically between the direct method and other methods, and as such it is unclear if our conclusions about time preferences are caused by the method of elicitation. It is thus recommended to replicate our study using an alternative time preference elicitation method that can accommodate negative discounting but is less susceptible to sequence effects [61, 62]. If our null result is replicated to other methods, this will provide a more reliable estimation of the lack of effect of this type of EFT treatment on time preference in life years.

In addition, we emphasize that the finding of “no significant effect” for EFT on cTTO utilities merely reflects insufficient evidence to reject a zero gap with the present sample size, standard deviations and chosen significance level. The assessment of EFT effectiveness also depends on the evaluative methods. For example, we could not declare EFT equivalent to either ERT or the ex-post direct adjustment on cTTO once MID bounds were applied. This is due to the difference in evaluation lens and two factors explain this outcome. First, equivalence hinges on the margin selected, and the Two One-Sided Tests framework in the equivalence analyses is more conservative than t-test/MER at the same significance level ($\alpha$). Second, modest sample size and larger standard deviations for more severe health states widen confidence intervals; these intervals can include zero (nonsignificance) yet still extend beyond the equivalence bounds, undermining equivalence claims. Therefore, defining a single universal equivalence margin is difficult given the sample variability inflates with severity of health state. Although EFT showed no significant effect, it is more important to assess the EFT effectiveness on cTTO utilities of different health states in clinical practice. Future research can employ larger, stratified samples and state-specific margins to claim the EFT effectiveness in practice.

Our study has several other limitations. First, although our sample size is comparable to or even larger than most similar studies in TTO health state valuation or EFT, the lack of significance in our findings regarding EFT could still be attributed to insufficient statistical power if the effect size is small. Furthermore, our small sample size may limit us to explore how different individuals vary in their response to EFT. For instance, some studies suggest that EFT reduces time preference more for younger adults than for older adults [43, 54]. However, in our study, EFT has no significantly different effect on cTTO utilities across different age groups (see Appendix 6). Another limitation could be that we conducted online video interviews rather than in-person interviews. Most previous EFT studies were conducted in controlled laboratory settings, which may provide a more suitable environment to imagine future episodic events [17, 18, 41, 46, 49, 55]. This may explain the lack of differences between the two arms, although existing evidence suggests that online video interviews do not seriously reduce data quality in TTO tasks [34, 51].

## Conclusion

This study is the first to investigate whether EFT can mitigate time preference and improve the health state valuation. While EFT has demonstrated its effectiveness in promoting farsighted decisions in varying domains, including health-related behaviors, our study reveals an insignificant effect of EFT on mitigating the distorting influence of time preference on cTTO utilities. Under the protocol implemented here, EFT does not appear to offer added value over existing time‑preference adjustment and to be an effective method for improving health state valuations. This lack of evidence highlights the need for further research to optimize time preference correction methods in cTTO valuations, potentially exploring other ex-post or ex-ante strategies that reduce (distortion related to) time preference.

## Figures and Tables

**Table 7:**
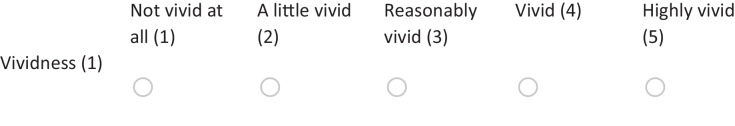
EFTvividness1 How vivid where your thoughts about the events you just described, i.e. your life and health in about 10 to 20 years from now?

**Table 8:**
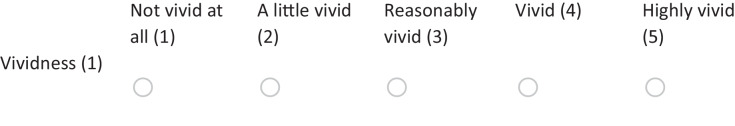
ERTVividness1 How vivid where your thoughts about the events you just described?

**Table 9:**
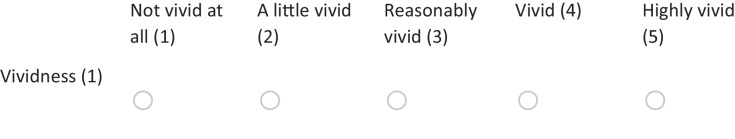
ERTVividness2 How vivid where your thoughts about the events you just described?

**Fig. 3:**
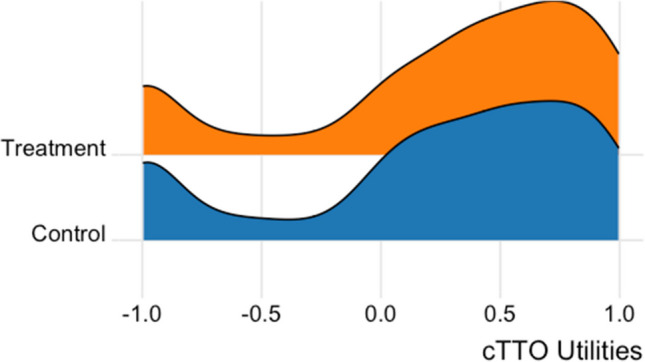
Ridgeline plot of cTTO utilities in both groups

**Fig. 4:**
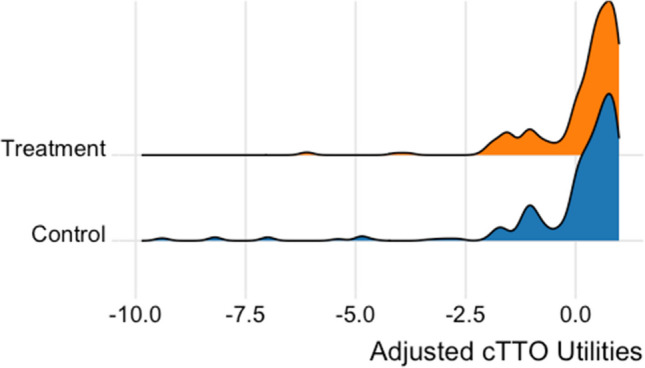
Ridgeline plot of adjusted cTTO utilities in both groups

**Fig. 5:**
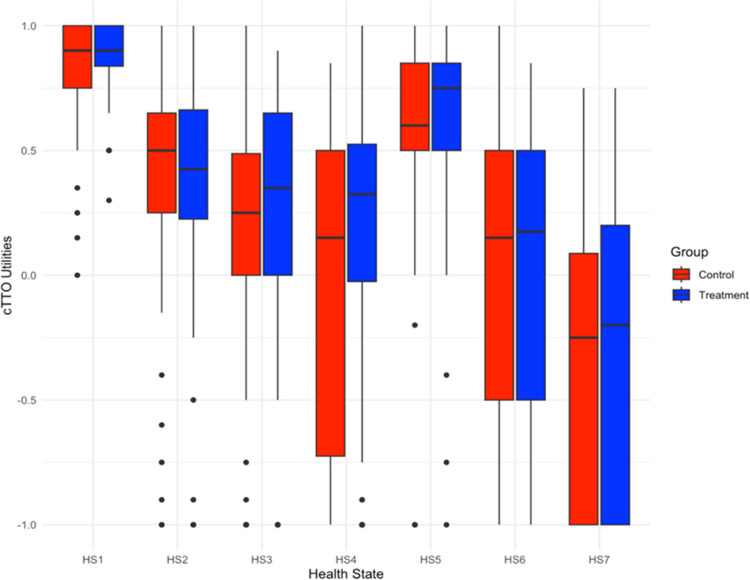
Boxplot of cTTO utilities across health states by control and treatment groups

**Fig. 6:**
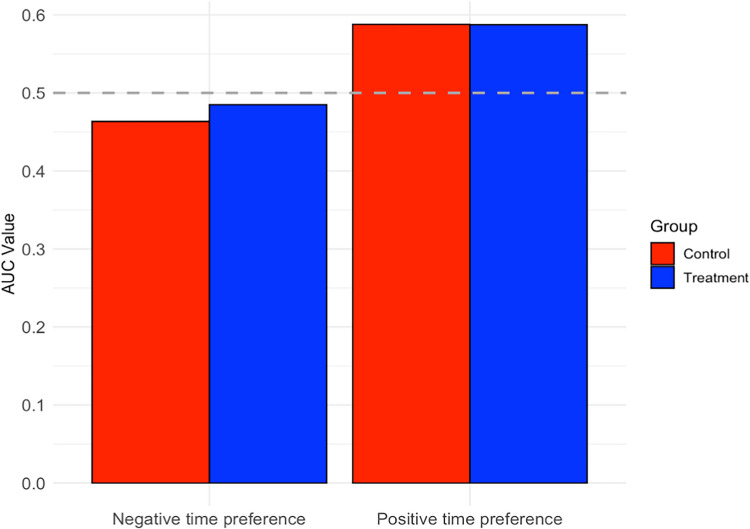
Average AUC values for control and treatment groups categorized by negative and positive time preference

## Data Availability

The data of this paper is available upon request.

## Appendix 1

The link to study design:

https://erasmusuniversity.eu.qualtrics.com/jfe/preview/previewId/c9421502-15fd-4078-9824-337692d2e94d/SV_3QX5ExfndgfnO5M?Q_CHL=preview&Q_SurveyVersionID=current.

**Questions on demographic variables:**

1. Education: What is the highest level of education you have completed?
  1. Some primary
  2. Completed primary school
  3. Some secondary
  4. Completed secondary school
  5. Vocational or similar
  6. Some university but no degree
  7. University bachelor’s degree
  8. Graduate or professional degree (MA, MS, MBA, PhD, JD, MD, DDS)
  9. Prefer not to say

To be noted: Education is further classified into lower education level: Some Primary, Completed Primary School, Some Secondary, Completed Secondary School; middle-level education: Vocational or Similar, Some University but no degree; higher education level: University Bachelor’s Degree, Graduate or professional degree (MA, MS, MBA, PhD, JD, MD, DDS).

2. Age: How old are you?
  1. Under 18
  2. 18-24 years old
  3. 25-34 years old
  4. 35-44 years old
  5. 55-64 years old
  6. 65+ years old
3. Gender: How do you describe yourself?
  1. Male
  2. Female
  3. Non-binary/third gender
  4. Prefer to self-describe
  5. Prefer not to say
4. Children: How many children under 18 live with you?
5. Marital status: What is your current marital status?
  1. Married
  2. Living with a partner
  3. Widowed
  4. Divorced/Separated
  5. Never been married
6. Religion: Would you describe yourself as religious?
  1. Yes
  2. No
  3. Other
7. Subjective life expectancy: How old do you expect to become (in years)?

**Self-reported health status using EQ-5D-5L:**

1. Please select the ONE box that best describes your health TODAY: MOBILITY

I have no problems in walking about

I have slight problems in walking about

I have moderate problems in walking about

I have severe problems in walking about

I am unable to walk about

2) Please select the ONE box that best describes your health TODAY: SELF-CARE

I have no problems washing or dressing myself

I have slight problems washing or dressing myself

I have moderate problems washing or dressing myself

I have severe problems washing or dressing myself

I am unable to wash or dress myself

3) Please select the ONE box that best describes your health TODAY: USUAL ACTIVITIES(e.g. work, study, housework, family or leisure activities)

I have no problems doing my usual activities

I have slight problems doing my usual activities

I have moderate problems doing my usual activities

I have severe problems doing my usual activities

I am unable to do my usual activities

4) Please select the ONE box that best describes your health TODAY: PAIN/DISCOMFORT

I have no pain or discomfort

I have slight pain or discomfort

I have moderate pain or discomfort

I have severe pain or discomfort

I have extreme pain or discomfort

5) Please select the ONE box that best describes your health TODAY: ANXIETY/DEPRESSION

I am not anxious or depressed

I am slightly anxious or depressed

I am moderately anxious or depressed

I am severely anxious or depressed

I am extremely anxious or depressed

**Self-reported health status using EQ-VAS:**

We would like to know how good or bad your health is TODAY. You will see a scale numbered from 0 to 100. 100 means the best health you can imagine. 0 means the worst health you can imagine.

## Appendix 2

1. EFT manipulation (treatment group)

Participants are instructed to imagine their lives in the next 10 and 20 years, specifically focusing on a particular health state (33333) described in the EQ-5D-5L system. They are asked to provide explicit descriptions of these imagined situations. Importantly, the durations of 10 and 20 years will be randomized to ensure variability. To initiate this exercise, participants are given an example to follow. The followings are the texts participants receive during the interview:

We would like to ask you to imagine your health in the future. Please imagine how your health will be in a specified period in the future and how it will affect your behavior. Try to describe this in as much detail as you can, as if it’s currently happening. We now first give you an example of how this might look like.

"In about 30 years, I will be married and have 3 grown-up children. I will live in a house in a village. I will have slight difficulties in walking and moderate problems in washing or dressing myself. I will have slight problems with my usual activities. I will have mild pain and other complaints. I will feel moderately anxious or depressed. I will still be capable of working, but only behind a desk, requiring assistance in several home and leisure activities. I cannot do sports anymore and have to rest frequently. I can walk medium distances but not too fast. You feel good in general, but your health problems have somewhat decreased your happiness.”

Instruction

Now, please consider yourself 10/20 years from now in the following health state:

- You have moderate problems walking about.
- You have moderate problems washing or dressing yourself.
- You have moderate problems doing your usual activities (e.g., work, study, housework, family, or leisure activities).
- You have moderate pain or discomfort.
- You are moderately anxious or depressed.

Now, consider yourself 20 years from now in the following health state:

- You have moderate problems walking about.
- You have moderate problems washing or dressing yourself.
- You have moderate problems doing your usual activities (e.g. work, study, housework, family, or leisure activities).
- You have moderate pain or discomfort.
- You are moderately anxious or depressed.

Can you describe how your life will be then, as if it’s currently happening? Please try to use a similar approach as in the example.

Interviewer then probes for further description by systematically asking the following elaboration questions (if not yet addressed by the respondent):

- “What will you be doing?”
- “Whom will you be with?”
- “Where will you be?”
- “How will you be feeling?”

Afterwards, they are asked,

How vivid where your thoughts about the events you just described? Not vivid at all, a little vivid, reasonably vivid, vivid, highly vivid.See Table 7.

2. ERT manipulation (control group)

We would now like to ask you to provide some details about a number of recent events. Please imagine the events we will ask you about Try to describe this in as much detail as you can, as if it’s currently happening. We now first give you an example of how this might look like.

“Just before your interview you went to the park with your friend Jane. You were at the local park, sitting by the waterfront eating lunch. You were admiring the beautiful scenery. You were feeling relaxed and enjoyed the nice weather outside. Some people with dogs as well as some runners passed by. After you finished your lunch, you went for a little walk along the trees. Then Jane and you said goodbye and both of you went home. Once you arrived at home you felt pleasurable and vital.”

Instruction

Now, please answer the following questions,

What did you have for breakfast this morning? Were you alone or with someone else? How did it taste? How were you feeling?

Can you describe how vivid where your thoughts about the events you just described? Not vivid at all, a little vivid, reasonably vivid, vivid, highly vivid. See Table 8.

What were you doing the last hour? Was it at home or somewhere else? Were you alone or together with other people? How were you feeling? Did you enjoy it?

Can you describe how vivid where your thoughts about the events you just described? Not vivid at all, a little vivid, reasonably vivid, vivid, highly vivid. See Table 9.

## Appendix 3

An example: how does direct method adjust time preference in the elicitation of cTTO utilities?

Employing direct method enables us to find several timing points T_d_, where L(T_1/4_)=0.25, L(_T1/2_)=0.5, L(T_3/4_)=0.75. Then we can use interpolation to derive the utility of other life durations in between (0,T_1/4_), (T_1/4,_T_1/2_), (T_1/2_,T_3/4_) or (T_3/4,_20). Suppose here is a person named Bob and we find that T_1/2_= 8, which L(8)=0.5 implies that for Bob, the utility of next 8 years is equivalent to the 12 years that follow it, i.e. L(T_1/2_=8)=L(20)- L(T_1/2_=8). It indicates that Bob has positive time preference since if there is zero time preference, a person should value same for the first 10 years and the next ten years, i.e. L(10)=0.5. Suppose we also find that T_1/4_= 3 and T_3/4_ = 12 for Bob. In the following, we provide two examples of how cTTO utilities can be adjusted for time preference using direct method in two scenarios, namely, better than death (BTD) and worse than death (WTD) health states, with those assigned values.

First, in a BTD health state, suppose Bob indicates Y = 7 in the comparison between health state Q and full health (FH), i.e. (Q, 10 years) ~ (FH, Y years) in the normal TTO task. In the conventional QALY which assumes no time preference, it implies U(Q) = 7/10 = 0.7 (eq. (2)). However, through the direct method discussed above, by interpolating between L(3) and L(8), we know for Bob, L(7) = 0.25 + (7-3)/(8-3) x 0.25 = 0.45. Similarly, L(10) can be interpolated between L(8) and L(12), which gives L(10) = 0.5 + (10-8)/(12-8) x 0.25 = 0.625 for Bob. Therefore, after adjusting the time preference, for Bob, U(Q) = L(7)/L(10) = 0.45/0.625 = 0.72, which is larger than the utility without considering time preference.

Second, in a WTD health state, health state utilities are elicited a lead-time TTO task. Suppose Bob indicates Y = 6 in the comparison between health state Q and FH, i.e. (FH, 10 years; Q, 10 years) ~ (FH, Y years). Similarly, in the conventional QALY which assumes no time preference, it implies U(Q) = (6-10)/(20-10) = -0.4 (eq. (4)). However, through the direct method, by interpolating between L(3) and L(8), we know for Bob, L(6) = 0.25 + (6-3)/(8-3) x 0.25 = 0.40. Besides, L(10) = 0.625 from the calculations above and L(20) = 1, after adjusting the time preference, for Bob, we obtain U(Q) = (0.40-0.625)/(1-0.625) = -0.6, which is lower than the utility without considering time preference.

We assume positive time preference in the above examples, which is the most common case in the real world. It implies that individuals often assign a lower weight to future years. Thus, it leads to downward bias for the cTTO utilities of BTD health states and upward bias for the cTTO utilities of the WTD health states without adjusting for time preference. Conversely, for cases with negative discounting, it results in the opposite pattern.

## Appendix 4

See Figs 3, 4 and 5.

## Appendix 5

See Tables 10 and 11.

**Table 10:** Mixed effects regression of utilities of better than dead observations, treatment group with other demographic variables

cTTO utilities	Coefficient (standard error)
Treatment (EFT)	0.044 (0.032)
Health state 11221	0.594 (0.028) ***
Health state 54231	0.282 (0.029) ***
Health state 34515	0.187 (0.029) ***
Health state 35245	0.181 (0.030) ***
Health state 33333	0.416 (0.028) ***
Health state 45144	0.118 (0.030) ***
Higher education	-0.055 (0.044)
Middle level education	-0.081 (0.052)
45-64 years	-0.014 (0.031)
65+ years	-0.021 (0.063)
Gender	0.007 (0.027)
Whether having children	-0.021 (0.033)
Marital status	0.073 (0.032) **
Religion	-0.093 (0.031) ***
EQVAS	-0.003 (0.001) ***
Subjective life expectancy	0.002 (0.001)
Vividness-1	-0.004 (0.016)
Vividness-2	0.015 (0.020)

* indicates significance level at 10%; **indicates significance level at 5%; *** indicates significance at 1%

**Table 11:** Mixed effects regression of utilities of worse than dead observations, treatment group with other demographic variables

cTTO utilities	Coefficient (standard error)
Treatment (EFT)	-0.051 (0.076)
Health state 11221	0 omitted
Health state 54231	0.228 (0.046) ***
Health state 34515	0.042 (0.038)
Health state 35245	0.039 (0.034)
Health state 33333	0.049 (0.067)
Health state 45144	0.098 (0.032) ***
Higher education	0.083 (0.096)
Middle level education	0.092 (0.105)
45-64 years	0.043 (0.077)
65+ years	0.123 (0.129)
Gender	0.078 (0.069)
Whether having children	0.031 (0.075)
Marital status	0.090(0.076)
Religion	-0.016 (0.076)
EQVAS	0.001 (0.002)
Subjective life expectancy	0.002 (0.003)
Vividness-1	0.055 (0.043)
Vividness-2	-0.079 (0.049)

*indicates significance level at 10%; **indicates significance level at 5%; ***indicates significance at 1%

## Appendix 6

See Tables 12, 13, 14 and 15.

**Table 12:** Mixed effects regression of utilities, treatment group with other demographic variables and the Interactions between age older than 45 and treatment group EFT

cTTO utilities	Coefficient (standard error)
Treatment (EFT)	0.081 (0.111)
Health state 11221	1.173 (0.042) ***
Health state 54231	0.696 (0.042) ***
Health state 34515	0.488 (0.042) ***
Health state 35245	0.361 (0.042) ***
Health state 33333	0.898 (0.042) ***
Health state 45144	0.325 (0.042) ***
Higher education	0.156 (0.097)
Middle level education	-0.073 (0.113)
Older than 45 years old	0.080 (0.091)
Gender	0.162 (0.062) ***
Whether having children	0.002 (0.072)
Marital status	0.151 (0.073) **
Religion	-0.130 (0.068) *
EQVAS	-0.004 (0.002) *
Subjective life expectancy	0.003 (0.003)
Vividness-1	-0.029 (0.037)
Vividness-2	-0.365 (0.309)
Older than 45 years old*Treatment	-0.141 (0.129)

*indicates significance level at 10%; **indicates significance level at 5%; ***indicates significance at 1%.

**Table 13:** Mixed effects regression of utilities, treatment group with other demographic variables and the Interactions between age older than 65 and treatment group EFT

cTTO utilities	Coefficient (standard error)
Treatment (EFT)	-0.000 (0.074)
Health state 11221	1.173 (0.042) ***
Health state 54231	0.696 (0.042) ***
Health state 34515	0.488 (0.042) ***
Health state 35245	0.361 (0.042) ***
Health state 33333	0.898 (0.042) ***
Health state 45144	0.325 (0.042) ***
Higher education	0.156 (0.097)
Middle level education	-0.080 (0.115)
Older than 65 years old	0.026 (0.165)
Gender	0.154 (0.062) **
Whether having children	-0.006 (0.074)
Marital status	0.150 (0.070) **
Religion	-0.132 (0.070) *
EQVAS	-0.004 (0.002) *
Subjective life expectancy	0.004 (0.003)
Vividness-1	-0.027 (0.037)
Vividness-2	-0.036 (0.043)
Older than 65 years old*Treatment	-0.198 (0.274)

*indicates significance level at 10%; **indicates significance level at 5%; ***indicates significance at 1%

**Table 14:** Mixed effects regression of utilities, treatment group with other demographic variables and the Interactions between EQVAS below 50 and treatment group EFT

cTTO utilities	Coefficient (standard error)
Treatment (EFT)	-0.037 (0.075)
Health state 11221	1.173 (0.042) ***
Health state 54231	0.696 (0.042) ***
Health state 34515	0.488 (0.042) ***
Health state 35245	0.361 (0.042) ***
Health state 33333	0.898 (0.042) ***
Health state 45144	0.325 (0.042) ***
Higher education	0.172 (0.097)
Middle level education	-0.065 (0.113)
45-64 years old	0.045 (0.070)
65 + years old	-0.022 (0.140)
Gender	0.160 (0.062) ***
Whether having children	-0.016 (0.074)
Marital status	0.128 (0.072) *
Religion	-0.117 (0.070) *
EQVAS below 50	0.088 (0.154)
Subjective life expectancy	0.002 (0.003)
Vividness-1	-0.022 (0.037)
Vividness-2	-0.044 (0.044)
EQVAS below 50*Treatment	0.280 (0.231)

*indicates significance level at 10%; **indicates significance level at 5%; ***indicates significance at 1%

**Table 15:** Mixed effects regression of utilities, treatment group with other demographic variables and the interactions between vividness and treatment group EFT

cTTO utilities	Coefficient (standard error)
Treatment (EFT)	0.012 (0.285)
Health state 11221	1.173 (0.042) ***
Health state 54231	0.696 (0.042) ***
Health state 34515	0.488 (0.042) ***
Health state 35245	0.361 (0.042) ***
Health state 33333	0.898 (0.042) ***
Health state 45144	0.325 (0.042) ***
Higher education	0.160 (0.098)
Middle level education	-0.073 (0.115)
45-64 years old	0.023 (0.071)
65 + years old	-0.025 (0.141)
Gender	0.150 (0.063) **
Whether having children	-0.005 (0.075)
Marital status	0.149 (0.073) **
Religion	-0.126 (0.070) *
EQVAS	-0.003 (0.002) *
Subjective life expectancy	0.004 (0.003)
Vividness-1	-0.045 (0.047)
Vividness-2	-0.016 (0.063)
Vividness-1*Treatment	0.055 (0.078)
Vividness-2*Treatment	-0.058 (0.091)

*indicates significance level at 10%; **indicates significance level at 5%; ***indicates significance at 1%

## Appendix 7

See Table 16, 17 and 18

**Table 16:** Mixed effects regression of utilities, treatment group with other demographic variables but only on the health state 33333 (what we used in the EFT questions)

cTTO utilities	Coefficient (standard error)
Treatment (EFT)	-0.014 (0.073)
Health state 33333	0.391 (0.046) ***
Higher education	0.163 (0.097) *
Middle level education	-0.067 (0.114)
45-64 years old	0.020 (0.070)
65 + years old	-0.032 (0.140)
Gender	0.155 (0.062) **
Whether having children	-0.006 (0.074)
Marital status	0.145 (0.073) **
Religion	-0.126 (0.070) *
EQVAS	-0.003 (0.002) *
Subjective life expectancy	0.003 (0.003)
Vividness-1	-0.025 (0.037)
Vividness-2	-0.040 (0.044)

*indicates significance level at 10%; **indicates significance level at 5%; ***indicates significance at 1%

**Table 17:** Mixed effects regression of utilities, treatment group with other demographic variables but only on the first health state (the declining effect with time passing by)

cTTO utilities	Coefficient (standard error)
Treatment (EFT)	0.049 (0.063)
The first experienced health state	0.156 (0.018)
Higher education	0.149 (0.101)
Middle level education	-0.056 (0.118)
45-64 years old	-0.104 (0.080)
65 + years old	-0.084 (0.143)
Gender	0.121 (0.066) *
Whether having children	-0.028 (0.080)
Marital status	0.167 (0.076) *
Religion	-0.154 (0.073) *
EQVAS	-0.004 (0.007)
Subjective life expectancy	0.002 (0.003)
Vividness-1	-0.007 (0.025)
Vividness-2	-0.006 (0.023)

*indicates significance level at 10%; **indicates significance level at 5%; ***indicates significance at 1%

**Table 18:** Mixed effects regression of utilities, treatment group with other demographic variables but only on the most severe health state (the declining effect with time passing by)

cTTO utilities	Coefficient (standard error)
Treatment (EFT)	-0.014 (0.073)
Health state 55555	-0.657 (0.042) ***
Higher education	0.163 (0.097) *
Middle level education	-0.067 (0.114)
45-64 years old	0.020 (0.070)
65 + years old	-0.032 (0.140)
Gender	0.155 (0.062) **
Whether having children	-0.006 (0.074)
Marital status	0.145 (0.073) **
Religion	-0.127 (0.070) *
EQVAS	-0.003 (0.002) *
Subjective life expectancy	0.003 (0.003)
Vividness-1	-0.025 (0.037)
Vividness-2	-0.040 (0.044)

*indicates significance level at 10%; **indicates significance level at 5%; ***indicates significance at 1%

## Appendix 8

See Fig. 6.

## Appendix 9: Power analysis and sample size decision

The analysis will compare the effect size between and EFT group and an ERT control using a two-tailed t-test for independent samples with equal allocation (1: 1).

According to Cohen d (Cohen, 1988), the expected treatment effect is, 

$$d={\textstyle\frac{{\overline X}_{EFT}-{\overline X}_{ERT}}{S_{pooled}}}$$

where

$$S_{pooled}=\sqrt{\textstyle\frac{\left(n_1-1\right)S_1^2+\left(n_2-1\right)S_2^2}{n_1+n_2-2}}$$

We apply Hedges’ adjustment to correct the bias caused by sample size,

$$J=1-{\textstyle\frac3{4df-1}},df=n_1+n_2-2$$

$$g=j\times d$$

If we expected the same size sample for each group,

$$n_{per\;group}={\textstyle\frac{2\left(Z_{1-\frac\alpha2}+Z_{1-\beta}\right)}{g^2}}$$

$\alpha$is the Type-I error (two-sided), which is typically chosen 0.05, leading to$Z_{1-{\textstyle\frac\alpha2}}=1.96,\beta$ is the power size, typically chosen 0.80, leading to$Z_{1-{\textstyle\beta}}=0.84$.

Therefore, if we expected Hedges’ g=0.52,the sample size per group will be around 59.

## Appendix 10

To determine whether EFT produces cTTO utilities that are practically indistinguishable from those obtained with ERT or with an ex-post time-preference adjustment, we conducted equivalence tests using the Two One‑Sided Tests (TOST) procedure (Lakens, 2017) as follows.

## 1. Hypotheses

We compared mean cTTO utilities between the ERT group and the EFT group (independent samples, equal variance assumption). The hypothesis set is, 

$$H_{01}:\theta\geq\pm\triangle\;and\;H_{01}:\theta\leq\pm\triangle$$

Where $\theta=\mu_{ERT}-\mu_{EFT},\mu$ is the mean cTTO utilities in the responding group.

We define that rejection of both nulls at per tail (combined 90 % CI) demonstrates practical equivalence.

## 2. Procedure

1. Choose and justify the equivalence margin

According to Walters & Brazier (2005) pooled 11 longitudinal datasets and found a mean MID for EQ-5D(-3L) of 0.074 utility units (ranging from –0.011 to 0.140), which indicate a difference smaller than 0.074 is considered clinically trivial. So, adopt +/-0.074 as our equivalence upper and lower bounds($\triangle_U$ and$\triangle_L$).

2. Calculate the pooled SD and standard error (SE)
  $$S_{pooled}=\sqrt{\textstyle\frac{\left(n_1-1\right)S_1^2+\left(n_2-1\right)S_2^2}{n_1+n_2-2}},SE=S_{pooled}\sqrt{{\textstyle\frac1{n_1}}+{\textstyle\frac1{n_2}}}$$
3. Calculate the t-statistic given the upper and lower bounds 
  $$\begin{array}{c}t_L={\textstyle\frac{\left({\overline X}_{EFT}-{\overline X}_{ERT}\right)-\triangle_L}{SE}},t_U={\textstyle\frac{\left({\overline X}_{EFT}-{\overline X}_{ERT}\right)-\triangle_U}{SE}}\\df=n_1+n_2-2\end{array}$$
  where ${\overline X}_{EFT}$and${\overline X}_{ERT}$ be be the mean cTTO utilities for each group, $n_1$and$n_2$ are corresponding sample size $S_1$and$S_2$ are their sample standard deviations.

Once having the t-statistic and its degrees of freedom *df,* the *p*-value is obtained from the cumulative distribution function (CDF) of the Student-t distribution.

## 3. Results

We conducted two separate equivalence tests for cTTO utilities. First, we compared the mean cTTO utilities between the EFT and ERT groups. Second, we compared the cTTO utilities in the EFT group with the time preference–adjusted cTTO utilities in the ERT group, to evaluate the relative effectiveness of EFT and direct method in adjusting for time preference. In both cases, the sample size for EFT group is$n_1=72\times7=504$, and for ERT group$n_2=78\times7=546$,given we have 7 health states.

## 3.1. Equivalence of cTTO utilities between the EFT and ERT groups

In this case, the mean cTTO utilities ${\overline X}_{EFT}$ and ${\overline X}_{ERT}$ are 0.268 and 0.215 in our study, and their sample standard deviations are $S_1=0.626$ and $S_2=0.640$. The results are shown in Panel A in Table 19.

**Table 19:** Equivalence tests of cTTO utilities: EFT vs. ERT and EFT vs. direct method

Outcome	Equivalence bound (Δ)	90 % CI for	*p*-value (≥+Δ)	*p*-value (≤−Δ)	*Equivalent?*
*Panel A:* cTTO utilities (EFT vs ERT)	0.074 (MID, Walters & Brazier (2005))	[–0.011, 0.117]	<0.001	0.296	No
	0.127 (Cohen’s *d* = 0.20 (“small”))	[–0.011, 0.117]	<0.001	0.030	Yes
*Panel B:* Utilities (EFT vs. direct method)	0.074 (MID, Walters & Brazier (2005))	[0.176, 0.417]	<0.001	> 0.999	No
	0.127 (Cohen’s *d* = 0.20 (“small”))	[0.176, 0.417]	<0.001	0.790	No

The observed mean difference is, $\theta_{obs}=\mu_{EFT}-\mu_{ERT}$

In Panel A, $$\theta_{obs}=0.053$$

In Panel B, $$\theta_{obs}=0.297$$

## 3.2. Equivalence between cTTO utilities in the EFT group and adjusted cTTO utilities in the ERT group

In this case, the mean cTTO utilities ${\overline X}_{EFT}=0.268$, and time preference adjusted utilities by direct method ${\overline X}_{ERT}=-0.0285$, and their sample standard deviations are $S_1=0.626$and $S_2=1.532$. The results are shown in the Panel B in Table 19.

## Footnotes

1 The EQ-5D-5L health states selected can represent a diverse spectrum of clinical conditions [68]. The review by Zhou et al. [68] provides clinical examples for different utility values. For example, the relatively good health state 11221, with a utility of approximately 0.871 according to the English tariff [20], corresponds to type-2 diabetes without complications. Similarly, moderately impaired states, such as 33333, reflect conditions like stable coronary artery disease or moderate COPD. More severe states include profiles such as 54231, corresponding to chronic spinal cord injury, and 34515, exemplified by severe chronic depression. The highly severe state 35245 aligns with metastatic cancer with severe pain, while 45144 corresponds to advanced Parkinson’s disease or severe haemophilia. Finally, the most severe state, 55555, represents conditions such as children with Morquio, which is a syndrome who rely entirely on wheelchair use.

2 Rosch et al. [50] found that EFT had a moderate effect (*Hedge’g* = 0.44, 95% CI [0.33, 0.55]) from 48 articles encompassing 174 effect sizes, including some unpublished articles. Ye et al. [67] meta-analyze 47 published articles with 63 effect sizes and found an EFT effect size with *Hedge’s g* = 0.52, 95% CI [0.42, 0.63].
