# Correction: Is episodic future thinking effective in mitigating the influence of time preference in time trade-off?

**DOI:** 10.1007/s10198-025-01862-8

**Published:** 2025-10-29

**Authors:** Zhongyu Lang, Liying Zhang, Stefan A. Lipman, Bradley Sugden, Kim Rand, Arthur E. Attema

**Affiliations:** 1 Erasmus School of Health Policy & Management, Erasmus University, P.O. Box 1738, Rotterdam, 3000 DR the Netherlands; 2 Erasmus Centre for Health Economics Rotterdam (EsCHER), Rotterdam, the Netherlands; 3 Department of Clinical Epidemiology and Medical Technology Assessment, Maastricht University Medical Centre, Maastricht, the Netherlands; 4 Health Services Research Centre, Akershus University Hospital, Akershus, Norway; 5 Math in Health B.V, Klimmen, the Netherlands

**Correction: The European Journal of Health Economics**

10.1007/s10198-025-01812-4

In this article the author’s name Bradley Sugden was incorrectly written as Bradely Sugden.

The original article has been corrected.

